# Designing Multifunctional Devices for Regenerative Pharmacology Based on 3D Scaffolds, Drug-Loaded Nanoparticles, and Thermosensitive Hydrogels: A Proof-of-Concept Study

**DOI:** 10.3390/pharmaceutics13040464

**Published:** 2021-03-30

**Authors:** Francesco Colucci, Vanessa Mancini, Clara Mattu, Monica Boffito

**Affiliations:** 1 Department of Mechanical and Aerospace Engineering, Politecnico di Torino, 10129 Turin, Italy; Colucci.fran@gmail.com (F.C.); V.Mancini@lumc.nl (V.M.); 2 Department of Anatomy & Embryology, Leiden University Medical Center, 2333 ZC Leiden, The Netherlands; 3 PolitoBIOMed Laboratory, Politecnico di Torino, 10129 Turin, Italy; 4 Institute for Chemical-Physical Processes, National Research Council (CNR-IPCF), 56124 Pisa, Italy

**Keywords:** thermosensitive hydrogels, nanoparticles, curcumin, scaffolds

## Abstract

Regenerative pharmacology combines tissue engineering/regenerative medicine (TERM) with drug delivery with the aim to improve the outcomes of traditional TERM approaches. In this work, we aimed to design a multicomponent TERM platform comprising a three-dimensional scaffold, a thermosensitive hydrogel, and drug-loaded nanoparticles. We used a thermally induced phase separation method to obtain scaffolds with anisotropic mechanical properties, suitable for soft tissue engineering. A thermosensitive hydrogel was developed using a Poloxamer^®^ 407-based poly(urethane) to embed curcumin-loaded nanoparticles, obtained by the single emulsion nanoprecipitation method. We found that encapsulated curcumin could retain its antioxidant activity and that embedding nanoparticles within the hydrogel did not affect the hydrogel gelation kinetics nor the possibility to progressively release the drug. The porous scaffold was easily loaded with the hydrogel, resulting in significantly enhanced (4-fold higher) absorption of a model molecule of nutrients (fluorescein isothiocyanate dextran 4kDa) from the surrounding environment compared to pristine scaffold. The developed platform could thus represent a valuable alternative in the treatment of many pathologies affecting soft tissues, by concurrently exploiting the therapeutic effects of drugs, with the 3D framework acting as a physical support for tissue regeneration and the cell-friendly environment represented by the hydrogel.

## 1. Introduction

Tissue engineering/regenerative medicine (TERM) is a multidisciplinary discipline aimed at designing functional constructs able to restore, maintain, and improve the functionality of damaged tissues or whole organs [1,2]. TERM has progressed greatly over the last few decades and currently includes many different facets while sharing some common ones, such as the use of specific “building blocks”. These can be (i) biomimetic materials mimicking the physicochemical and mechanical properties of a native environment, (ii) 3D networks in the form of scaffolds or hydrogels providing physical support and guidance for new tissue formation and organization, (iii) stem cells or specialized cells, and (iv) signaling cues in the form of physical (e.g., mechanical and electrical cues) or biochemical (e.g., growth factors) stimuli [3,4].

Among the different building blocks of TERM approaches, three-dimensional matrices and their forming materials strongly influence the structural and functional integration of implanted constructs into the host tissue/organ [4]. For instance, the success rate of TERM approaches targeting the regeneration of soft anisotropic tissues, such as the skeletal muscle and the heart tissue, strongly rely on the use of elastomeric polymers and their processing into structurally and mechanically anisotropic scaffolds [5,6]. Moreover, many publications have demonstrated that, for successful cardiac TERM, scaffolds with pores aligned along a preferred direction exhibit better cell organization and functionality compared to randomly oriented ones. Indeed, cells cultured on anisotropic scaffolds more easily adopt a stretched and rod-like morphology, exhibit more organized sarcomeres, and overexpress end-to-end gap junctions [7,8].

Since the first use of the expression “regenerative pharmacology” in 2007, many approaches have been developed to combine drug delivery with TERM to accelerate, guide, and optimize the development, maturation, and recovery of functionality of bioengineered tissues/organs [9,10]. Pharmaceutical compounds in the form of small drug molecules or biomolecules such as growth factors are signaling cues that provide biochemical stimuli for repairing and regenerating tissues/organs [8].

To properly exert their function, such compounds require integration with the other building blocks of TERM under mild conditions to avoid potential degradation/denaturation [11,12]. Prolonged, sustained, and localized delivery of these molecules from TERM constructs is desired to enable proper guidance of the regenerative process according to a well-defined time schedule, to limit off-targets effects, and to minimize adverse events or side effects [12].

To this aim, surface coating of 3D scaffolds with drug molecules or biomolecules can be achieved through a simple impregnation procedure or through direct entrapment into scaffolds or hydrogels during their fabrication [13,14,15,16,17,18]. Direct payload loading into composite films of chitosan and poly(allylamine hydrochloride) was proposed by M. Sohail Sarwar et al. [19]. Loading into 3D matrices has also been reported from scaffolds based on the silicate 1393 glass coated with a mesoporous bioactive glass loaded with dexamethasone and bone morphogenic protein-2 for infected bone treatment [14]. In another work, Kayıran Çelebier et al. directly loaded naproxen sodium into the pore walls of poly(lactide-*co*-glycolide) (PLGA) scaffolds prepared using the emulsion freeze-drying technique for corneal epithelium regeneration [20]. Similarly, Dai and colleagues directly loaded doxorubicin into poly(lactic acid)/pearl electrospun nanofibrous membranes for tumor treatment [15]. Lin and Chang encapsulated diclofenac into an alginate hydrogel, which was then 3D bioprinted into multilayered constructs that were finally coated with chitosan to modulate the release kinetics of the encapsulated drug [21].

Besides the possibility to directly integrate active molecules into 3D frameworks, therapeutic agents can also be loaded into scaffolds or hydrogels upon previous encapsulation into *ad hoc* designed nano- or micro-carriers, such as polymeric or inorganic particles [22,23]. This approach has the advantage of protecting the payload from fast degradation/deactivation and enhances control over its release kinetics. For instance, we recently demonstrated that ibuprofen loading into mesoporous particles before encapsulation into thermosensitive hydrogels allows for prolonged and sustained delivery compared to free drug directly loaded into the gel [24,25]. Similar particles were also successfully incorporated into poly(lactic acid)/polyaniline electrospun scaffolds [26]. He and colleagues recently described the loading of growth factor-containing particles into a temperature-responsive hydrogel for the selective delivery of biomolecules inducing vascularization processes [27]. PLGA micro- and nanoparticles loaded with curcumin and bovine serum albumin were also successfully incorporated into gelatin scaffolds cross-linked with glutaraldehyde [28]. Nooeaid et al. recently reported on the design and characterization of porous gelatin membranes loaded with PLA particles releasing tetracycline hydrochloride, which exhibited enhanced physical and thermal stability compared to pristine scaffolds, antibacterial properties, and compatibility with human dermal fibroblasts [29]. In a previous publication by our group, we incorporated simvastatin-loaded PLGA microparticles into porous freeze-dried chitosan-gelatin scaffolds and analyzed the effects of carrier concentration on the physical properties of the porous matrices; the release profile of the drug; and the viability, proliferation, and osteoblastic differentiation of a clonal human osteoblastic cell line [30]. We also used gelatin containing indomethacin-loaded particles as the coating material of bioactive inorganic scaffolds for bone tissue engineering, demonstrating that the coating improved the compressive strength of the porous matrices and progressively released the drug up to 7 days without any negative effect on viability and alkaline phosphatase activity of human osteoblast-like cells [31]. In the same year, Ferreira et al. demonstrated the possibility to incorporate similar polymeric particles into PLA scaffolds fabricated via thermally induced phase separation with the aim to develop biomimetic constructs for wound healing and soft tissue regeneration [32]. Very recently, PLGA particles encapsulating doxorubicin hydrochloride were added to a calcium phosphate bone cement, resulting in a more effective and localized delivery of the cargo compared to direct drug loading into the cement as such [23].

In the abovementioned research, particles were directly embedded or absorbed into hydrogels or scaffolds during fabrication of the device. With this approach, particles may be subjected to harsh preparation conditions (e.g., solvents, temperature gradients, and mechanical stresses) needed to fabricate the device, therefore altering their integrity and drug encapsulation/release properties. Moreover, uneven particles distribution into the scaffold structure may result in poor uniformity of drug release from the system [33].

Therefore, in this work, we aimed to design a multicomponent device containing nanoparticles, in which the particles are not directly embedded in the scaffold structure but are finely dispersed within a hydrogel vehicle that carries the drug-loaded polymeric particles into the pores of a three-dimensional polymer construct to further improve the potential and versatility of regenerative pharmacology approaches. The overall goal of this system is to provide a versatile structure for the regeneration of soft tissues that encompasses adequate mechanical support, a cell-friendly environment and proper nutrient trafficking for cell homing, and drug release capacity.

**Figure 1:**
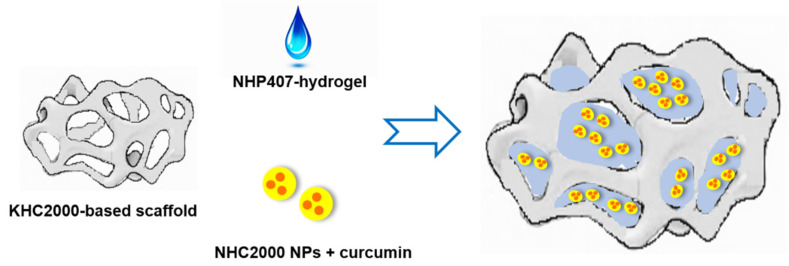
Schematic representation of the multicomponent system.

To achieve this aim, we developed a multifunctional patch resulting from the assembly of three main constituents (Figure 1): (i) an anisotropic porous scaffold fabricated by the thermally induced phase separation (TIPS) technique starting from a customized poly(ester urethane) with elastomeric properties [34], (ii) a thermosensitive hydrogel based on an *ad hoc* synthesized poly(ether urethane) with improved thermal gelation and stability in a watery environment compared to commercially available formulations based on similar amphiphilic polymers [35], and (iii) curcumin-loaded poly(ester urethane)-based nanoparticles prepared by the single emulsion nanoprecipitation method [36].

Each constituent was first characterized as a single entity through physicochemical and morphological analyses. Finally, they were combined into a single device, thus providing a proof of concept on the possibility to engineer multifunctional patches able to simultaneously provide physical support to the regenerating tissue; a prolonged and sustained release of therapeutic agents; and a watery environment that provides modulation of payload release and a cell-friendly milieu into the inner scaffold cavities, which often suffer poor cell colonization. We showed that hydrogel embedding favored absorption of a model molecule of nutrients (i.e., fluorescein isothiocyanate dextran 4kDa) from the surrounding watery environment, thus providing more favorable conditions for scaffold homing and colonization by cells. Thus, based on its mechanical properties, cell-friendly milieu, and drug release ability, the scaffold warrants further investigation in soft tissue engineering applications of skeletal and heart muscles, where stretchable constructs are required [5,6,37,38].

## 2. Materials and Methods

### 2.1. Materials

Poly(ε-caprolactone) (PCL) diol (${\bar{M}}_{n}$ 2000 g/mol), Poloxamer^®^ P407 (P407, ${\bar{M}}_{n}$ 12,600 g/mol, 70% poly(ethylene oxide) (PEO)), 1,6-hexamethylene diisocyanate (HDI), N-Boc-serinol, L-lysine ethyl ester (H-Lys(H)-OEt) dihydrochloride, and dibutyltin dilaurate (DBTDL) were purchased from Sigma Aldrich, Milan, Italy. *N*-Boc-serinol and l-lysine ethyl ester were used as chain extenders during poly(urethane) (PU) synthesis. Poly(ε-caprolactone) diol and Poloxamer^®^ P407 were dried under reduced pressure at 100 °C for 8 h and then cooled down at 45 °C under vacuum to remove residual water before use. HDI was distilled under reduced pressure before use. For nanoparticle preparation, curcumin, poly(vinyl alcohol) (PVA), and Tween^®^ 80 were purchased from Sigma Aldrich, Milan, Italy. All solvents were purchased from Sigma Aldrich, Milan, Italy, in analytical grade. Milli-Q deionized water was produced by a Millipore water purification system (Millipore Corporation, Milan, Italy).

### 2.2. Poly(urethane) Synthesis and Characterization

#### 2.2.1. Synthesis Protocol

The two PCL-based poly(urethane)s (acronyms KHC2000 and NHC2000) used in this work were synthesized following a two-step synthesis procedure in inert atmosphere, as previously described [34]. Dried PCL diol was first dissolved in anhydrous 1,2-dichloethane (DCE) at 20% *w*/*v* concentration at 80 °C. The diisocyanate was then added to the solution (2:1 molar ratio with respect to PCL diol), using DBTDL as a catalyst and reacted with the macrodiol at 80 °C for 150 min, followed by the addition of the chain extender (*N*-Boc-serinol or l-lysine ethyl ester) (3% *w*/*v* in DCE) at 1:1 molar ratio with respect to the macrodiol upon cooling of the system at room temperature. For H-Lys(H)-OEt, triethylamine (Sigma Aldrich, Milan, Italy) was also added. The chain extension reaction was stopped after 16 h by adding methanol. The poly(urethane) was then collected by precipitation of the polymer solution in petroleum ether (4:1 volume ratio with respect to DCE volume) and purified twice by dissolution in *N*,*N*-dimethylformamide (DMF, 20% *w/v*) followed by precipitation in methanol (5:1 volume ratio with respect to DMF volume). The obtained powder was finally dried under vacuum at 40 °C for 72 h.

The protocol used for the synthesis of the water-soluble poly(ether urethane) (acronym NHP407) was similar to that adopted for the synthesis of the poly(ester urethane)s and in accordance with Boffito et al. [39]. In this case, the triblock copolymer Poloxamer^®^ P407 was used as a macrodiol, HDI was used as a diisocyanate, and *N*-Boc serinol was used as a chain extender. This synthesis procedure differed from the previous one only in the second step that was carried out at 60 °C for 90 min. Purification was carried out by precipitating the polymer solution in DCE (20% *w*/*v*) into a mixture of diethyl ether and methanol (98:2 *v:v*) (5:1 volume ratio with respect to DCE). The collected polymer was then washed in diethyl ether (5 g × 100 mL) overnight and collected through a Büchner funnel. The polymer was finally dried overnight under vacuum at room temperature, ground, and kept in a nitrogen atmosphere at 5 °C.

#### 2.2.2. Poly(urethane) Nomenclature

PU nomenclature was based on the nature of the constituent segments, as summarized in Table 1. The first letter indicates the chain extender: K corresponds to l-lysine ethyl ester, and N corresponds to *N*-Boc-serinol; the second letter H indicates the diisocyanate HDI, C2000 refers to PCL-diol with number average molecular weight ${\bar{M}}_{n}$ 2000 Da, and P407 corresponds to Poloxamer^®^ 407 used as a macrodiol.

#### 2.2.3. Chemical Characterization

Attenuated Total Reflectance Fourier Transform Infrared (ATR-FTIR) spectra of the three synthesized poly(urethane)s were recorded at room temperature (16 scans at 4 cm^−1^ resolution) in the spectral range from 4000 to 600 cm^−1^ using a Perkin Elmer Spectrum 100 (Wahtham, MA, USA) equipped with an ATR accessory (UATR KRS5) with diamond crystal. Each spectrum was analyzed using the Perkin Elmer Spectrum Software.

Number average and weight average molecular weight (${\bar{M}}_{n}$ and ${\bar{M}}_{w}$), and molecular weight distribution ($D=\;{\bar{M}}_{w}/{\bar{M}}_{n}$) of the synthesized PUs were estimated by Size Exclusion Chromatography (SEC) (Agilent Technologies 1200 Series, Santa Clara, CA, USA) using tetrahydrofuran (inhibitor-free, CHROMASOLV^®^ Plus, for HPLC, ≥99.9%, Romil, Cambridge, UK) as the mobile phase according to a previously reported protocol [39]. ${\bar{M}}_{n}$, ${\bar{M}}_{w}$, and D were determined by the Agilent ChemStation Software relative to a calibration curve based on 10 narrow polystyrene standards ranging in ${\bar{M}}_{n}$ from 740 to 18 × 10^4^ g/mol.

### 2.3. Scaffold Fabrication and Characterization

#### 2.3.1. Scaffold Fabrication through Thermally Induced Phase Separation (TIPS)

KHC2000-based porous scaffolds were fabricated by Thermally Induced Phase Separation (TIPS) and subsequent solvent extraction according to a previous protocol with slight modifications [40]. Briefly, the poly(urethane) was first solubilized at 70 °C in dimethyl sulfoxide (DMSO, Sigma Aldrich, Milan, Italy) at a final concentration of 12% *w/v*; then, the obtained solution was poured into stainless steel parallelepiped molds (35 × 20 × 15 mm) and cooled down at −80 °C for 3 h. The quenching was performed under application of a thermal cooling gradient to induce DMSO crystal growth, i.e., pore formation, in a preferred direction. To this aim, all mold walls were insulated using a thermal insulating material and cotton wool except one. To extract DMSO, the frozen scaffolds were placed for 2 days in a water/ethanol solution (30:70 *v/v*), which was refreshed twice a day. The scaffolds were finally freeze-dried (Martin Christ ALPHA 2-4 LSC, Osterode am Harz, Germany), snap-frozen in liquid nitrogen, and cut to obtain matrices with a thickness of about 1 mm.

#### 2.3.2. Scaffold Characterization

##### Morphology and Porosity Measurements

The morphology of the produced scaffolds was evaluated by Scanning Electron Microscopy (SEM; LEO 1450VP). Micrographs were taken with a beam voltage of 20 kV and magnifications of 50× and 100×. Both cross and longitudinal sections of the scaffold were observed. Samples were sputter-coated with gold before analysis. Image data were imported into ImageJ software for analysis. The average pore size and pore size distribution were obtained by measuring the diameter of 80 randomly chosen pores.

Scaffold porosity was determined by the liquid displacement method, using ethanol as a displacement liquid [41]. Each tested scaffold was first weighed (W_1_) and then immersed in a glass cylinder containing a known volume of ethanol. The sample was pressed to force air out of the scaffold until no air bubbles were seen, allowing the ethanol to fill the pores. The ethanol-impregnated scaffold was then removed from the cylinder and weighed again (W_2_). The percentage porosity (*p*%) of the scaffold was determined by the following equation (Equation (1)).

(1) $$p\%=\;\frac{W_{2}-\;W_{1}}{W_{2}}\;\cdot 100$$

The results are reported as mean ± standard deviation of 3 measurements.

##### Contact Angle Measurements

The wettability of the scaffold was characterized by means of a contact angle measure instrument CAM 200 (KSV Instrument, Ltd., Helsinki, Finland) using a sessile drop method in advancing mode. PU films fabricated by solvent casting were also analyzed as a control condition. A drop of distilled water (5 µL) was gently deposited onto the surface of the sample, and one image was recorded immediately after deposition. Each recorded image was analyzed using the Attension Theta software that allows for the automatic curve fitting of the drop profile based on the Young and Laplace equations. The results are reported as mean ± standard deviation of 3 measurements.

##### Hydrolytic and Enzymatic Degradation Tests

Hydrolytic and enzymatic degradation tests (Lipase from porcine pancreas, Sigma Aldrich, Milan, Italy) were performed on round (6 mm diameter) scaffolds. For hydrolytic degradation, the samples were placed in vials containing 0.1 mL of phosphate buffered saline (PBS, pH 7.4) per milligram of PU scaffold. For enzymatic degradation, a lipase concentration of 0.3 mg/mL was used. Both degradation tests were carried out at 37 °C, and the degradation medium was renewed every 3 days. Once a week and once a month for enzymatic and hydrolytic degradation tests, respectively, three samples were withdrawn, washed with distilled water, and dried at 37 °C until a constant weight was reached. At each time interval, the residual weight (%) of the specimens was evaluated according to Equation (2).

(2) $$residual\;weigh\;\left(\%\right)=100-\;\frac{W_{0}-\;W}{W_{0}}\;\cdot 100$$

where *W*_0_ is the initial weight of the sample and *W* is its weight after degradation at a particular time interval, both measured by a microbalance. At each timepoint, the collected samples were also analyzed by SEC according to the previously described protocol to evaluate changes in PU molecular weight and molecular weight distribution.

The results are reported as mean ± standard deviation of 3 measurements.

##### Mechanical Properties

Rectangular samples (15 × 5 × 1.5 mm) were mechanically characterized by stress–strain tests performed using a MTS QTest/10 Elite Controller equipped with a 10 N load cell. The cross-head speed was 2 mm/min. The tests were conducted at room temperature in both dry and wet conditions (wet samples were obtained by incubating the scaffolds in water at room temperature overnight). The mechanical properties were characterized in both the longitudinal and cross directions. The results are reported as mean ± standard deviation of 3 measurements.

### 2.4. Hydrogel Preparation and Characterization

Thermosensitive hydrogels were prepared with NHP407 at a previously optimized concentration of 10% *w/v* in PBS [39]. Polymer solubilization was carried out at 5 °C overnight to avoid micellization and/or gelation during solution preparation.

The sol-to-gel transition of aqueous NHP407 solutions was investigated by tube-inverting test in temperature ramp and isothermal conditions according to the protocol reported by Pontremoli et al. [25]. NHP407 samples (1 mL) were prepared according to the previously described procedure in Bijou sample containers (Thermo Scientific™ Sterilin™, Milan, Italy). Tube-inverting test in temperature ramp conditions was performed in the temperature range between 6 and 40 °C to assess the lower critical gelation temperature (LCGT). The test was conducted through a step-by-step temperature increase, each step consisting of a 1 °C temperature increase followed by temperature maintenance for 5 min and vial inversion for visual inspection of the sol and gel phases. Sol and gel states were defined based on the presence of flow along vial walls within 30 s of sample inversion.

Hydrogel gelation time was studied by incubating the samples at 37 °C. The sol-to-gel transition was verified by inverting the vials at predefined timepoints of 1, 2, 3, 4, 5, 6, 7, and 8 min for 30 s. The conditions of sol and gel were defined as “flow liquid sol” and “no flow solid gel” within the 30 s of observation, respectively.

Rheological measurements were carried out on a stress-controlled rheometer (MCR302, Anton Paar GmbH, Graz, Austria) using a 50 mm parallel plate geometry. The rheometer was equipped with a Peltier system for temperature control. Small Amplitude Oscillatory Shear (SAOS) tests were performed to characterize the viscoelastic properties (frequency sweep tests, frequency range from 0.1 to 100 rad/s, strain = 0.1%, 37 °C) and the yield stress (strain sweep tests, frequency = 10 Hz, strain from 0.01 to 500%, 37 °C). Frequency sweep tests were carried out also at 25 and 30 °C to assess if the hydrogel was a sol, a biphasic system, or a completely developed gel at each predefined temperature. For each analysis, the sample was put on the lower plate of the rheometer at 0 °C, heated at the required temperature, maintained in quiescent conditions for 10 min to reach thermal stability, and finally isothermally tested. Finally, temperature ramp tests at 2 °C/min and constant shear rate (1 Hz) were performed to obtain information about the temperature-driven sol–gel transition (temperature ranging from 0 to 40 °C).

### 2.5. Hydrogel-Loaded Scaffolds

#### 2.5.1. Assembly Procedure

To evaluate the capability of the porous scaffolds to be combined with the hydrogel, 30 µL of NHP407-based sol-gel system (10% *w/v* concentration) was deposited on the surface of KHC2000 scaffolds (round samples with 6 mm diameter and 1.5 mm thickness) and the time required for complete absorption was evaluated by visual inspection. The whole procedure was carried out at 5 °C to keep the hydrogel in the sol state and to facilitate scaffold impregnation.

#### 2.5.2. Nutrient Permeability Test

Permeability studies were performed to model the exchange of nutrients throughout the hydrogel-impregnated scaffolds. Fluorescein isothiocyanate dextran (M_w_ 3000–5000 g/mol; Sigma-Aldrich, Milan, Italy; FD4) is generally used as a model of nutrients [42], since its Stokes radius (14 Å) is higher than that of nutrients (glucose and NaCl show a Stokes radius of 3.8 and 1.4 Å, respectively). KHC2000 scaffold samples loaded with NHP407-based sol–gel systems were put in a plastic vial and incubated at 37 °C for 10 min to induce the sol-to-gel transition. Then, 1 mL of a FD4 solution in PBS (1 mg/mL) previously conditioned at 37 °C was added to each vial and the samples were incubated at 37 °C (IKA KS-4000i). At predefined time steps (2, 5, 24, and 48 h), 3 samples were taken and the residual absorbance of the FD4 solution was measured by UV-VIS spectroscopy (UV/VIS spectrophotometer Lambda 365 from Perkin Elmer^®^, Waltham, MA, USA) in the 350–600 nm range, since the main absorption intensity peak of FD4 appears at 490 nm. The amount of FD4 absorbed by each specimen was defined as the difference between the starting and the residual FD4 content in the solution incubated with the samples. The amount of FD4 in each sample was calculated by referring to a calibration curve based on FD4/PBS standards with well-defined concentrations in the range 0.05–0.4 mg/mL. Scaffolds not loaded with the hydrogels were also characterized to assess the effect of scaffold impregnation with the hydrogels on nutrient permeability. The test was conducted in triplicate. The results are reported as mean ± standard deviation.

### 2.6. Nanoparticle Preparation and Characterization

#### 2.6.1. Preparation of nanoparticles (NPs)

Polyurethane nanoparticles were prepared with NSHC2000 by a single emulsion solvent extraction evaporation technique, as previously described by Mattu et al. with some modifications [36].

Briefly, 100 mg of the polymer was dissolved in ethyl acetate (4 mL) and added dropwise into 4 mL of water containing 2.5% *w/v* Tween 80 and sonicated for 2 min. The primary emulsion was added into 50 mL of water containing 1% *w/v* PVA and homogenized at 13,500 rpm. After solvent evaporation, particles were collected by centrifugation and washed three times with distilled water.

Curcumin-loaded NPs (1% and 5% *w/w* with respect to the polymer weight, namely 1 mg and 5 mg of drug) for in vitro drug release studies were prepared according to the above-described procedure by adding the drug to the polymer solution in ethyl acetate.

#### 2.6.2. NP Characterization

Average particles size was determined by dynamic light scattering technology (Nanoseries, Nano-ZS; Malvern Instruments, Malvern, UK). The amount of encapsulated curcumin was quantified by UV analysis. Measurements were carried out in triplicate.

For evaluation of curcumin loading efficiency, the freeze-dried NPs were dissolved into 4 mL ethyl acetate to extract the encapsulated drug. After ethyl acetate evaporation, 10 mL of ethanol was added to selectively dissolve curcumin. The solution was then filtered through a 0.45 µm polyvinylidene fluoride (PVDF) membrane and analyzed by UV spectrometry at an absorbance wavelength of 439 nm (UV/VIS spectrophotometer Lambda 365 from Perkin Elmer^®^, Waltham, MA, USA).

Drug encapsulation efficiency (EE%) was determined according to Equation (3).

(3) $$EE\%=\;\frac{W_{cur_{d}}}{\;W_{cur_{t}}}\cdot 100$$

where *W_curd_* is the weight (mg) of the drug detected by UV and *W_curt_* is the theoretical amount of drug expected in the NPs (i.e., the amount initially provided).

For the determination of curcumin release profiles, NPs were incubated at 37 °C in the release solution. The amount of drug released was measured after 1 h and 3 h followed by daily measurements until complete release was achieved. At the predetermined timepoints, the release solution was centrifuged at 10,500 rpm for 15 min to collect the NPs. The release medium was withdrawn and freeze dried, while NPs were resuspended in fresh medium. The released drug was measured by adding ethanol to the freeze-dried release solution, followed by drug detection by UV-Vis. Samples were analyzed in triplicate.

#### 2.6.3. Antioxidant Activity of Released Curcumin

The antioxidant activity of curcumin was determined using DPPH (2,2-diphenyl-1-picrylhydrazyl) as a free radical. This method is generally used to assess the DPPH• free radical scavenging capacity of antioxidant drugs [43,44]. The DPPH radical absorbs at 517 nm, but its absorption decreases upon reduction by an antioxidant or a radical species. When a hydrogen atom or electron is transferred to the odd electron in DPPH•, the absorbance at 517 nm decreases proportionally to the increase in non-radical forms of DPPH.

The amount of released curcumin from the 1- and 4-day timepoints was tested. Released curcumin was solubilized in 20 µL of methanol and added to 980 µL of a 0.25 mM methanol DPPH• solution. Control samples without curcumin and comparison samples with free curcumin at equivalent concentrations to the release solutions were also prepared. The decrease in absorbance was determined at 517 nm after 30 min incubation in the dark. The percentage inhibition of the DPPH radical was calculated according to Equation (4).

(4) $$\%\;Inhibition=\;\left(1-\frac{A_{s}}{A_{c}}\right)*100$$

where *A_s_* is the absorbance of the analyzed samples containing free or released curcumin and *A_c_* is the absorbance of the control samples.

### 2.7. Preparation and Characterization of NP-Loaded Hydrogels

NPs were added to the prepared hydrogels in the sol state (at 5 °C) to a final concentration of 20 mg NPs/mL of hydrogel solution. Rheological characterization of NP-loaded hydrogel was performed as described above for the un-loaded hydrogel system.

For release studies, NP-loaded hydrogels (20 mg NP/mL) were prepared in Bijou sample containers according to the previously described protocol [22]. Prior to the tests, all samples were incubated at 37 °C for 15 min to form a gel. Then, 1 mL of distilled water previously conditioned at 37 °C was added to each gel and the vials were kept at 37 °C in an incubator (IKA KS-4000i). The amount of drug released was measured after 1 h and 3 h, and then daily until complete dissolution of the gel. At predetermined timepoints, water was removed from the vials and the same volume of fresh water at 37 °C was added. The samples were-freeze dried to collect the released curcumin that was quantified by UV-Vis analysis (UV/VIS spectrophotometer Lambda 365 from Perkin Elmer^®^) after solubilization in ethanol. All tests were conducted in triplicate. The results are reported as mean ± standard deviation.

### 2.8. Statistical Analysis

The results are reported as mean ± standard deviation of three samples. Statistical analysis was performed using GraphPad Prism version 5.03 for Windows (GraphPad Software, La Jolla, CA, USA; www.graphpad.com accessed on 22 March 2021). *T*-Test analysis with a 95% confidence interval was used for comparisons.

## 3. Results and Discussion

### 3.1. Poly(urethane) (PU) Synthesis

**Figure 2:**
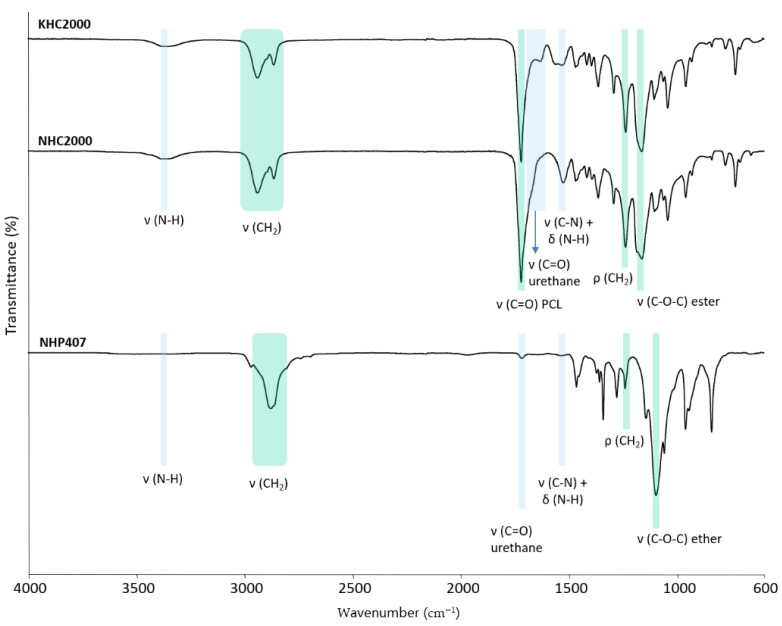
Attenuated Total Reflectance Fourier Transform Infrared (ATR-FTIR) spectra of KHC2000, NHC2000, and NHP407 poly(urethane)s. Light blue bars identify the characteristic peaks of newly formed urethane domains, and green bars refer to the typical bands of poly(ε-caprolactone) (PCL) and P407.

PUs were successfully synthesized as confirmed by Attenuated Total Reflectance Fourier Transform Infrared spectroscopy (Figure 2). Indeed, the ATR-FTIR spectra of KHC2000, NHC2000, and NHP407 poly(urethane)s showed the typical absorption peaks of newly formed urethane bonds: the peaks in the region between 1620 and 1640 cm^−1^ can be ascribed to the stretching vibration of the carbonyl groups, while the peak at *ca.* 3330 cm^−1^ represents the N–H stretching vibration [34,45]. The peak around 1535 cm^−1^ results from the concurrent N–H bending and C–N stretching of urethane domains. The absence of signals at 2200 cm^−1^ proved the complete conversion of isocyanate groups during polymer synthesis. The successful incorporation of PCL and P407 building blocks into KHC2000, NHC2000, and NHP407 polymer chains was demonstrated by the appearance of their characteristic absorption peaks in the registered ATR-FTIR spectra. In detail, in the ATR-FTIR spectra of both KHC2000 and NHC2000, the peaks at 1720 and 1160 cm^−1^ can be correlated with the stretching vibrations of carbonyl and C–O–C linkages of PCL, respectively [34]. Differently, in the ATR-FTIR spectrum of NHP407, the absorption band around 1100 cm^−1^ is attributed to the –OCH_2_CH_2_ units of PEO blocks of P407 [45]. Absorption peaks within 2860 and 2940 cm^−1^ and at *ca.* 1235 cm^−1^ come from –CH_2_ stretching and rocking vibrations, respectively.

Finally, SEC analyses further demonstrated the successful synthesis of high molecular weight poly(urethane)s, as summarized in Table 2, which reports the number average molecular weight ($\bar{M_{n}}$) and polydispersity index ($D=\bar{M_{w}}/\bar{M_{n}})$ data. The low polydispersity index values measured by SEC indicated a narrow distribution of the molecular weight and thus good control over the synthesis process.

### 3.2. Scaffold Characterization

#### 3.2.1. Morphology, Porosity, and Surface Characterization

**Figure 3:**
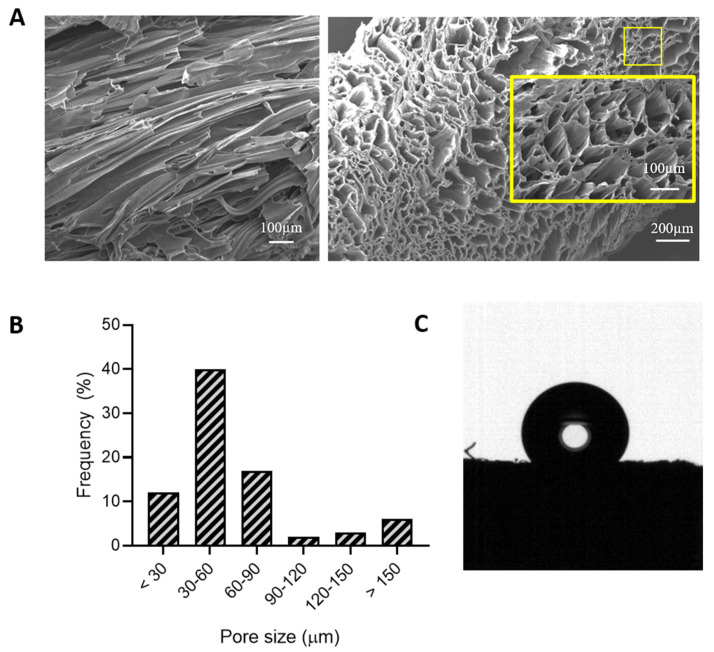
(**A**) SEM micrographs of KHC2000 scaffolds analyzed in the longitudinal and transverse directions (left and right, respectively), (**B**) pore size distribution obtained by analyzing SEM images through ImageJ software, and (**C**) image of a water drop deposited on a KHC2000 scaffold surface during static contact angle measurement.

Scaffold morphology was evaluated by SEM (Figure 3A). In detail, both longitudinal and cross sections were analyzed to verify the successful production of porous matrices with the desired architecture. Scaffolds exhibited a porous microarchitecture, with open and interconnected pores. In addition, scaffold pores turned out to be elongated and stretched according to the direction of the applied cooling gradient during matrix fabrication. Such pore organization is suitable for the repair and regeneration of anisotropic muscle and cardiac tissues, allowing for better morphological and functional integration of the implanted scaffolds into the host milieu, and proper guidance for cell arrangement and differentiation [7,8]. Scaffold pore size distribution was evaluated using ImageJ (Figure 3B), evidencing the presence of pores with a mean size ranging between tens and hundreds of micrometers (minimum and maximum measured diameters were 15 and 330 µm), which are reported to favor both vascularization, and cell homing and migration [6].

Scaffold porosity measured by the liquid displacement method was 76 ± 1%. The static contact angle was measured to be 125.8 ± 1.4°, which is representative of a hydrophobic surface (Figure 3C). The contact angle value measured for the fabricated 3D porous scaffolds was significantly higher (*p* < 0.0001) with respect to one of the KHC2000 films (Table 2). This behavior can be correlated with the aligned fiber-like structure and the high degree of order at both the micro- and nano-scales characterizing the developed scaffolds [46].

#### 3.2.2. Hydrolytic and Enzymatic Degradation

**Figure 4:**
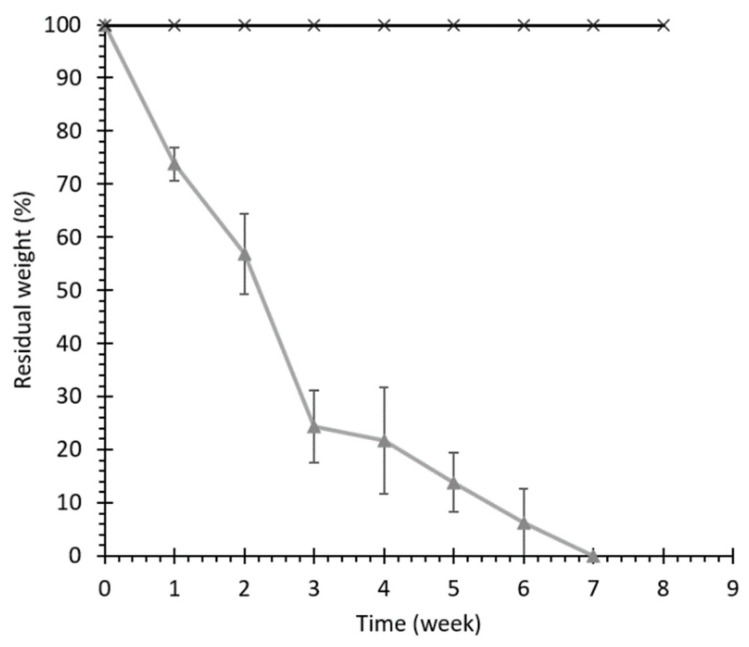
Percentage of residual weight of KHC2000 scaffolds during hydrolytic (black) and enzymatic (grey) degradation.

The hydrolytic degradation properties of KHC2000 scaffolds were evaluated in physiological-like conditions, i.e., in PBS at 37 °C. Accelerated hydrolytic degradation of KHC2000 scaffolds was studied by adding lipase to the degradation medium, which has been demonstrated to selectively catalyze the hydrolysis of the ester bonds in the PCL segments [47]. Figure 4 reports the percentage of residual weight of samples measured at different timepoints during hydrolytic and enzymatic degradation. Weight loss during enzymatic degradation proceeded faster than hydrolytic degradation, which did not cause any significant weight change up to 8 weeks of incubation time. These results are in agreement with previous observations on 3D-printed scaffolds based on a poly(ester urethane) of similar composition [48]. However, in this work, complete enzymatic degradation of the scaffold was achieved after 7 weeks while the 3-printed matrices degraded faster (3 weeks). Such a difference can be probably ascribed to the different structures of the matrices rather than to differences in polymer chemical compositions (the two materials differed only in the diisocyanate used during synthesis, i.e., 1,4 butane diisocyanate and 1,6-hexamethylene diisocyanate). TIPS-fabricated scaffolds have a more intricate internal architecture and poor wettability. Thus, they likely exposed a reduced surface area to lipase action, which accounts for the slower degradation. Indeed, lipase-mediated degradation is known to proceed through surface erosion, while hydrolytic degradation is known to proceed through a bulk degradation mechanism [48].

At each timepoint during enzymatic degradation, SEC analysis was also performed on the residual KHC2000 scaffolds, evidencing a progressive molecular weight decrease over time up to complete sample degradation (approximately 55% of number average molecular weight lost after three weeks of incubation). Conversely, no relevant changes in molecular weight were observed in samples collected during hydrolytic degradation, in agreement with the absence of any changes in sample mass during incubation.

#### 3.2.3. Mechanical Properties

Scaffold mechanical characterization was conducted through tensile tests performed under dry and wet conditions at room temperature. Moreover, analyses under dry conditions were carried out in both the longitudinal (i.e., parallel to the stretched pores) and cross directions to evaluate scaffold anisotropy. The measured mechanical parameters (i.e., Young’s modulus, and strain and stress at break) are summarized in Table 3.

**Figure 5:**
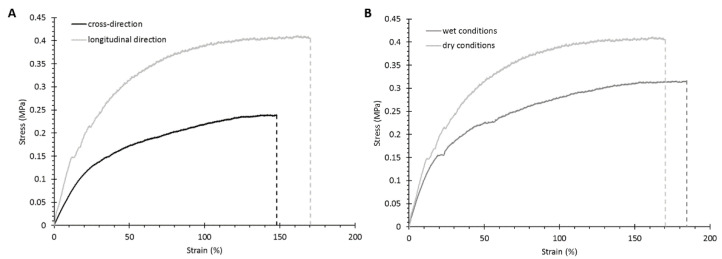
Stress–strain curves for KHC2000 scaffolds analyzed in different conditions: (**A**) in the cross-sectional and longitudinal directions in the dry state and at room temperature, and (**B**) in the longitudinal direction in wet and dry conditions at room temperature.

The measured mechanical parameters in the longitudinal and cross-sectional directions proved that the fabricated matrices exhibited anisotropic mechanical properties in addition to the structural anisotropy observed by SEM: Young’s modulus, and strain and stress at break decreased (significant decrease for Young’s Modulus value, *p* = 0.0203) when the samples were characterized in the cross-sectional direction, i.e., in the transverse direction compared to the pore main direction. Figure 5A reports the typical trends of the stress–strain curves measured in the longitudinal and cross directions, highlighting that the scaffolds mechanically behaved in a similar way irrespective of the testing direction, with an initial elastic deformation followed by plastic behavior until failure.

The fabricated scaffolds were also mechanically characterized in wet conditions to replicate the physiological environment (Table 3 and Figure 5B). Due to the plasticizing effect of water, both Young’s modulus and the stress at break values decreased under wet conditions while the strain at break increased (significant difference in Young’s modulus, *p* = 0.0362). These findings are consistent with our previous published works reporting the mechanical characterization of different polymeric substrates [40,49,50,51].

### 3.3. Hydrogel Characterization

**Figure 6:**
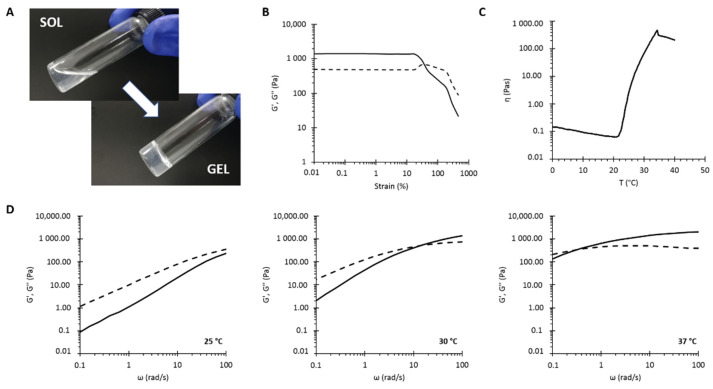
NHP407 hydrogel (10% *w*/*v* polymer concentration) characterization through the tube-inverting test (**A**) and rheology: (**B**) trends of storage (G’, black continuous line) and loss (G″, black dashed line) moduli as measured during strain sweep test at 37 °C, (**C**) trend of viscosity (η) as a function of temperature (T) during the temperature ramp test, and (**D**) trends of storage (G′, black continuous line) and loss (G″, black dashed line) moduli as measured during the frequency sweep test at 25, 30, and 37 °C.

NHP407 hydrogel at 10% *w*/*v* concentration was first qualitatively characterized by the tube-inverting test (Figure 6A) in temperature ramp conditions within 6 and 40 °C to define its lower critical gelation temperature (LCGT) and in isothermal conditions to estimate its gelation time at 37 °C. The hydrogel LCGT value was estimated to be around 32 °C, while gelation at 37 °C, defined by the absence of sample flow along vial walls during inversion, was measured to be 7 min. These results are in complete agreement with our previously published data [39]. Hydrogel thermal gelation and mechanical properties were also investigated through rheological tests (Figure 6B–D). The strain sweep test characterized the sample’s mechanical properties as a function of the applied strain at physiological temperature (Figure 6B), evidencing that the NHP407 hydrogel exhibited a linear viscoelastic behavior under the application of strain values below 18.6% (γ_L_). Indeed, at higher deformation, the storage modulus (G′) trend started to decrease while the loss modulus (G″) slightly increased, which is typical of gel networks where microcracks begin to form as a consequence of the applied strain. Under the application of 35% strain, the sample underwent complete mechanical failure (γ_gel-sol_), as evidenced by the G′/G″ crossover, with G″ becoming higher than G′, which is typical of fluid systems. The temperature-dependent sol-to-gel transition of NHP407 hydrogel was instead characterized by temperature ramp test within the 0–40 °C temperature range (Figure 6C) and frequency sweep tests at 25, 30, and 37 °C (Figure 6D). The trend of viscosity as a function of temperature evidenced an initial decrease with increasing temperature, characteristic of fluid systems, until a minimum value of viscosity (0.15 Pa·s initial viscosity vs. 0.06 Pa·s minimum value of viscosity) was reached at 20.7 °C, which marked the beginning of the gelation process (T_onset_). Then, a monotonic viscosity increase was observed because of micelle nucleation and packing into a well-organized network. At temperatures higher than 34.4 °C, the viscosity started to decrease due to gel crumbling resulting from its incapability to withstand the application of a continuous strain rate [45].

Frequency sweep tests evidenced the progressive temperature-guided transition of a NHP407-based system from the sol to the gel phases. At 25 °C the registered trends of G′ and G′′ as a function of angular frequency were characteristic of fluid systems (G′′ > G′), while at 30 °C, the hydrogel behaved as a biphasic system in which the sol and gel phases coexist, as evidenced by the presence of a crossover point between G′ and G′′ (ω_CROSS_) (at 10 rad/s) within the analyzed frequency range. With further temperature increase at 37 °C, the crossover point between G′ and G′′ moved towards lower values as a result of the progressive transition from the sol to the gel phases. At 37 °C, the crossover point between G′ and G′′ was at 0.33 rad/s, indicating that the system was in an incipient gel state. Nonetheless, the NHP407 sample was not a fully developed gel at 37 °C, since the shear moduli were not frequency independent.

### 3.4. Nanoparticle Characterization

**Figure 7:**
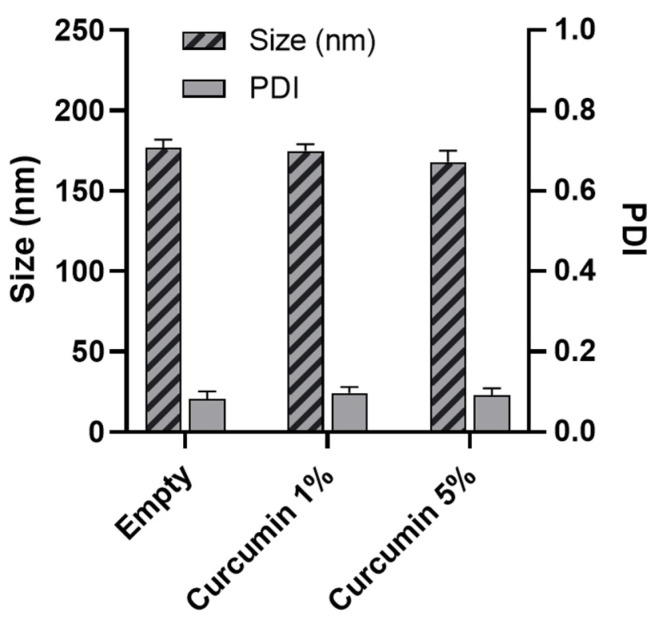
Size and polydispersity index (PDI) of empty and curcumin-loaded nanoparticles.

NPs with small size and low polydispersity index (PDI) were successfully obtained with NHC2000 (Figure 7). NPs had an average size of 177 nm with a PDI of 0.08. This is coherent with our previous reports, where we designed NHC2000 NPs for the release of paclitaxel, achieving a small size of 180 nm for empty particles [36]. When loaded with curcumin (1% and 5% *w/w*), the size of the NPs remained small with a slight reduction (Figure 7), indicative of high affinity between the polymer and the drug.

**Figure 8:**
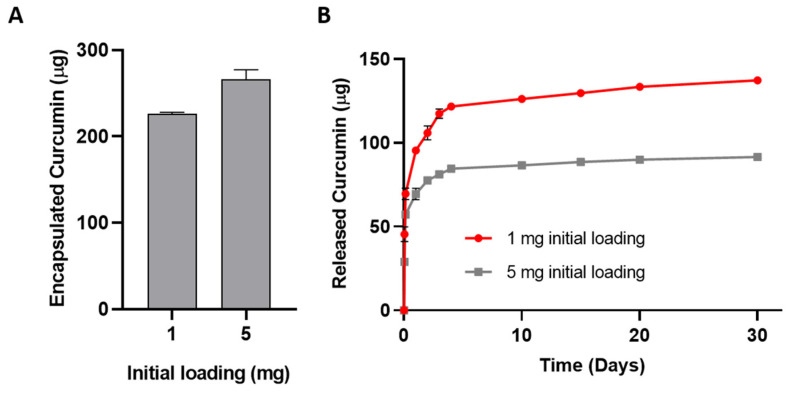
Encapsulation efficiency of curcumin inside NPs (**A**) and drug release profiles (**B**) evaluated for NPs prepared at different drug inputs.

The encapsulation efficiency, defined as the percentage of drug effectively loaded into NPs compared to the initial drug input (e.g., 1% and 5% of the polymer weight, namely 1 mg and 5 mg initially loaded curcumin), was evaluated as a function of the initial input. Successful curcumin encapsulation was achieved with high efficiency, as high as 20% for the 1 mg-loaded formulation, corresponding to 225 µg of drug (Figure 8A). The 5 mg-loaded formulation led to a much lower encapsulation efficiency of ≈5%, corresponding to 260 µg of loaded drug. Thus, increasing the amount of drug input in the formulation from 1 to 5 mg resulted in a significantly lower EE and in nearly the same amount of drug loaded inside the NPs. This indicates that saturation of the loading capacity was achieved already at 1% loading, similar to previous reports on curcumin-loaded chitosan NPs [52,53] and to our previous findings with gold nano-constructs loaded within PLGA NPs [54].

The curcumin release profile shows a faster release from 1%-loaded formulation, which reached nearly 60% of the encapsulated dose after 30 days of incubation and about 35% after the first day of incubation. On the other hand, the 5%-loaded formulation displayed slower release, with a smaller dose of curcumin released at each timepoint and only nearly 30% of drug release at the 30-day timepoint (Figure 8B).

This sustained release from the 1%-loaded formulation supports our findings that polyurethane NPs can prolong drug release [55]. Indeed, most literature reports indicate faster curcumin release. For instance, Anitha et al. reported a 74% release in 5 days from chitosan NPs [52] while thermo-responsive polymer micelles were shown to release curcumin in temperature- and pH-dependent fashion, reaching 80% release after 12 h below the lower critical solution temperature of the polymer at pH 4 [56].

Our results indicated that 1% loading was enough to achieve high entrapment capacity of the drug and that this formulation was able to release more drug in a sustained fashion. Therefore, the 1%-loaded formulation was selected for further testing.

**Figure 9:**
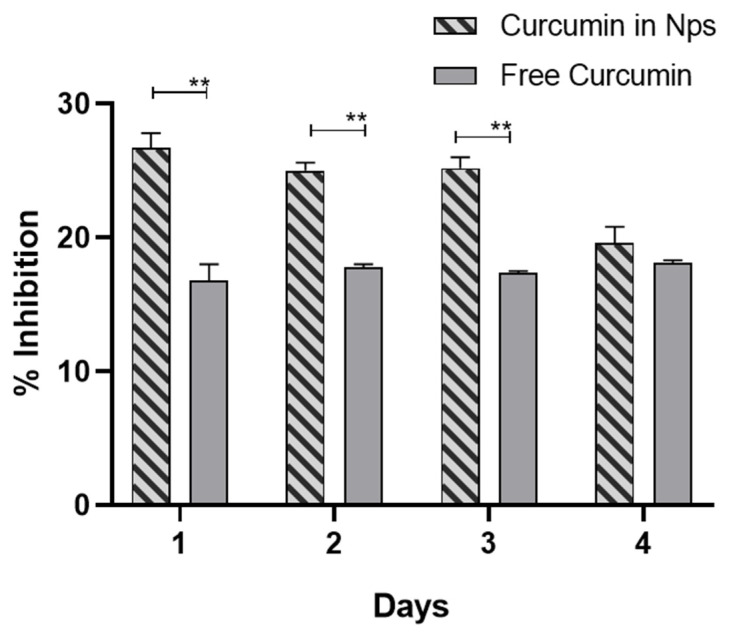
Antioxidant activity of free curcumin and curcumin released from NPs measured by DPPH (2,2-diphenyl-1-picrylhydrazyl) reduction assay. Significant results: *p* = 0.000459, *p* = 0.000039, *p* = 0.000074 for the results on day s1, 2, and 3, respectively (*T*-test). ** *p* < 0.01.

The antioxidant activity of the drug released form NPs (Figure 9) was significantly higher than that of free curcumin at the same dose.

The DPPH solution shows a strong absorption band at 517 nm, which is reduced proportionally to the degree of reduction achieved. The remaining DPPH, measured after 30 min of incubation, can be correlated to the radical scavenging activity of the antioxidant [57]. Our results indicate that the encapsulation of curcumin does not alter the drug effect. On the contrary, the antioxidant activity is enhanced with respect to the free drug, suggesting protection of the payload upon encapsulation in the polymer matrix.

### 3.5. Characterization of Hydrogel Embedded with Nanoparticles

#### 3.5.1. Study of the Sol-to-Gel Transition

**Figure 10:**
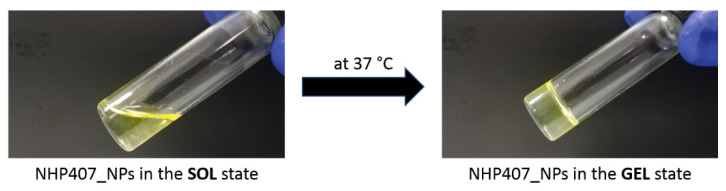
Appearance of the NHP407_NPs hydrogel in the sol state at low temperature (5 °C) and in the gel state upon incubation at 37 °C.

The sol-to-gel transition of the NHP407 hydrogel (10% *w/v* concentration) loaded with NPs at 20 mg/ml_hydrogel_ (NHP407_NPs) was studied through both qualitative and quantitative characterizations. Table 4 summarizes the main properties of NHP407 and NHP407_NPs hydrogels evaluated through tube-inverting and rheological tests. Figure 10 shows the appearance of NHP407_NPs hydrogel in the sol and gel states upon incubation at 37 °C.

The temperature-induced sol-to-gel transition of NHP407 hydrogel turned out to be slightly affected by particle encapsulation, although no detrimental effects were observed. Indeed, gelation temperature decreased to 29 °C while no differences were observed in the qualitative evaluation of gelation time at physiological temperature. However, from a quantitative point of view, particle inclusion inside the hydrogel phase mainly affected its response to applied deformation and its viscosity. Indeed, particle-loaded hydrogel showed an improved resistance to applied strain, with critical and break deformation values (i.e., γ_L_ and γ_gel-sol_, respectively) significantly higher with respect to the control sample not loaded with NPs. Regarding viscosity, particle addition to the hydrogel induced a *ca.* 2-fold increase in initial and minimum values of viscosity measured during the temperature ramp test. Additionally, the temperature that marks the beginning of the sol-to-gel transition (T_onset_) decreased to 19.4 °C in NHP407_NPs, suggesting that the particles allow the system to initiate its transition from sol to gel at a lower temperature, in agreement with the lower LCGT value evaluated by the tube-inverting test. Nonetheless, particle loading did not evidently affect the kinetics of the sol-to-gel transition, as evidenced by frequency sweep tests and gelation time tests at 37 °C. The obtained results clearly suggest that the particles co-participate in the sol-to-gel transition of NHP407 hydrogel. In particular, the behavior of the NHP407_NPs hydrogel is completely in agreement with our recently published observations on the thermal gelation and mechanical properties of similar poly(ether urethane)-based hydrogels loaded with mesoporous carbons in the presence of sodium dodecyl sulfate (SDS) acting as a dispersant of the inorganic phase into the polymeric phase [24]. The poly(ester urethane) forming the nanoparticles most likely interacted with the polymeric chains forming the hydrogel through hydrogen bonds, which co-participate together with hydrophobic forces to micelle nucleation and aggregation [24,58]. A similar behavior was also observed in poly(ether urethane)-based hydrogels loaded with silica-particles coated with a pH-sensitive self-immolative polymer [59].

#### 3.5.2. Drug Release Profile

**Figure 11:**
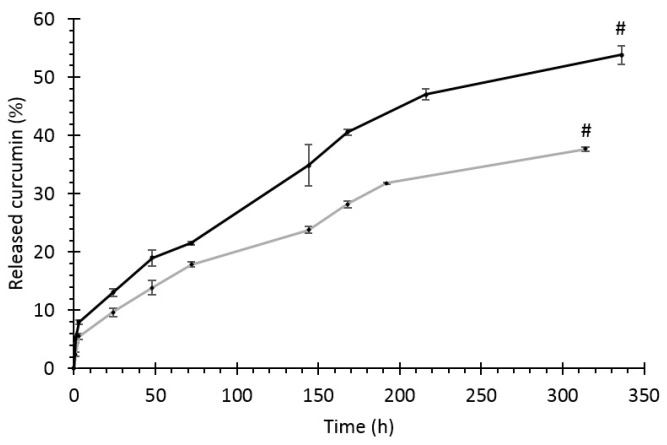
Curcumin release profile from the hydrogel and from NPs embedded in the hydrogel. The symbol # identifies the timepoint of complete sample dissolution.

The drug release profile from the hydrogel or from the particles embedded in the hydrogel (Figure 11) was measured until complete dissolution of the gel matrix (which takes place between days 12 and 13).

At each timepoint, the detected curcumin is higher for the NP-containing hydrogel. This is probably linked to particle diffusion from the hydrogel matrix [22] and to drug degradation/oxidation. Indeed, curcumin is sensitive to the degradative environment losing its antioxidant activity, as also demonstrated by the DPPH assay (Figure 9).

### 3.6. Assembly of Multifunctional NHP407_NPs-Loaded Scaffolds

#### 3.6.1. Hydrogel Absorption by KHC2000 Scaffolds

**Figure 12:**
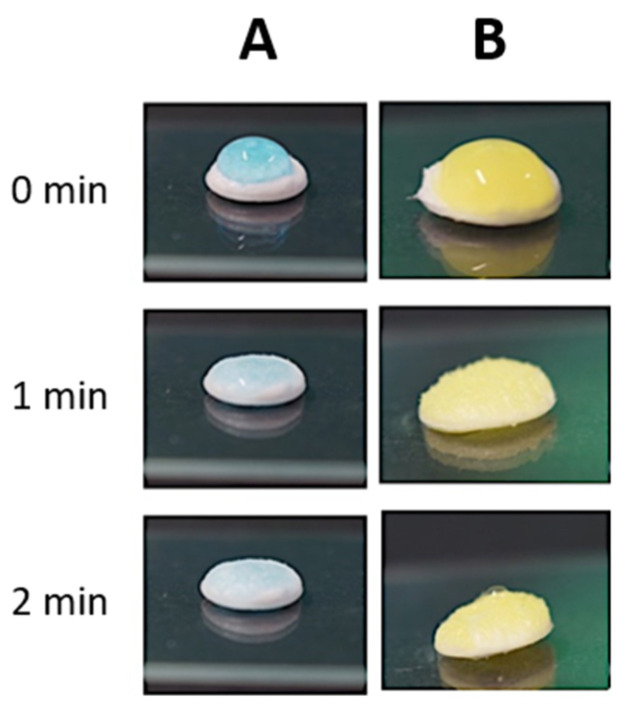
Absorption of NHP407-based hydrogels by KHC2000 scaffolds: (**A**) hydrogel not loaded with the particles and (**B**) hydrogel loaded with NPs.

The capability of the scaffolds to absorb the hydrogel was documented with photographs taken at 1 and 2 min after hydrogel deposition on the scaffold’s surface (Figure 12). The loading of NPs into the hydrogel did not affect its capability to be absorbed by the scaffold. Indeed, in all the investigated conditions, complete absorption of the hydrogel phase by the scaffolds was achieved within 2 min after deposition.

#### 3.6.2. Permeability Test

**Figure 13:**
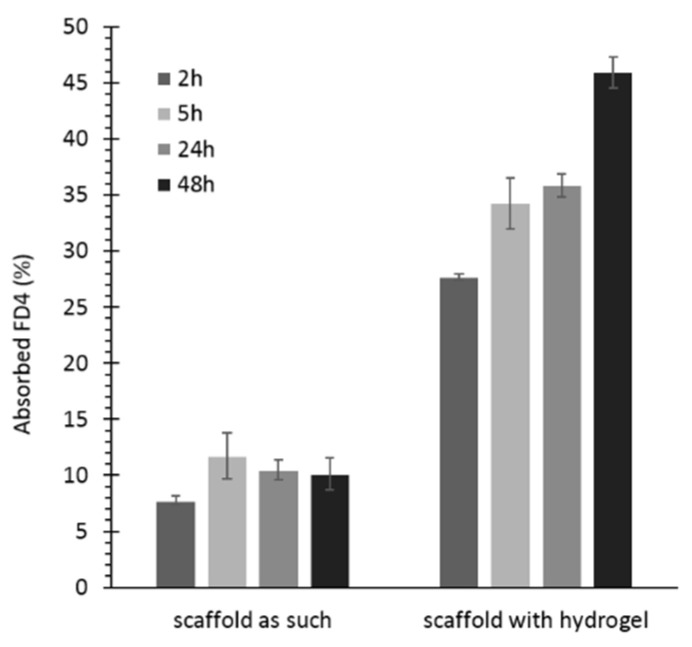
Percentage of absorbed FD4 by KHC2000 scaffolds as such and loaded with NHP407 hydrogel, measured at different timepoints (i.e., at 2, 5, 24, and 48 h of incubation). Significant results: *p* < 0.0001, *p* = 0.0002, *p* < 0.0001, *p* < 0.0001 for the results at 2, 5, 24, and 48 h, respectively (*T*-test).

Permeability studies were conducted to model nutrient transport through the scaffold and to evaluate the potential contribution of the hydrogel to this key process in the perspective of scaffold colonization by cells. Fluorescein isothiocyanate-dextran ($\bar{M_{w}}$ 3000–5000 g/mol, FD4) was selected as a model of nutrients, [42] since its Stokes radius (14 Å) is higher than that of nutrients (glucose and NaCl have a Stokes radius of 3.8 and 1.4 Å, respectively). Figure 13 reports the percentage of FD4 absorption by KHC2000 scaffolds as such and loaded with the NHP407 hydrogel.

KHC2000 control samples showed lower permeability to FD4 because of their high hydrophobicity and the absence of an internal reservoir able to carry FD4 molecules. The NHP407 hydrogel instead acted as a reservoir for FD4 upon its absorption by KHC2000 scaffolds, resulting in significantly higher percentages of absorbed FD4 at each timepoint. Moreover, while in virgin scaffolds FD4 absorption did not change over time, remaining constant at approximately 10%, in NHP407 hydrogel-loaded scaffolds, the percentage of FD4 molecules absorbed by the device increased over time, reaching ca. 46% after two days of incubation. These results suggest that the developed multifunctional matrices could effectively act as a reservoir of nutrients available for the encapsulated and/or recruited cells.

## 4. Conclusions

We reported a multicomponent drug delivery system made of three main compartments, namely (i) a porous scaffold with elastomeric properties and anisotropic structure; (ii) a thermo-responsive, water soluble hydrogel; and (iii) curcumin-loaded polymer NPs. This system exploits polymer NPs to deliver curcumin in a controlled fashion and to preserve its activity by protecting the payload from degradation [28,60]. To achieve this, we exploited a thermo-responsive hydrogel to embed the particles under mild conditions and to transport them uniformly and in intact form inside the pores of the scaffold. Our results show that the developed thermosensitive hydrogel retained its gelation properties even upon particle embedding (sol-to-gel transition at physiological temperature within 7 min) and was easily integrated into the intricated porosity of the fabricated anisotropic scaffolds within 2 min post-deposition on their surface. Moreover, the loading of the particles into the hydrogel phase did not negatively affect their release potential. The polymeric particles also provided a protective environment for curcumin from degradation [61,62]. This allows us to preserve the advantages of using NPs, such as controlled release and protection of the active principle, while maintaining the benefits of using a porous scaffold, such as support for tissue regeneration. For instance, the mechanical and structural anisotropy as well as the measured mechanical properties are compatible with repair and regeneration of damaged soft anisotropic tissues, such as the skeletal muscle and the heart tissue, where highly stretchable constructs with stiffnesses within tens of kilopascals and a few megapascals are required [5,6,37,38]. Thus, this drug delivery device may find useful application for localized drug delivery, where support for tissue regeneration is also needed. Moreover, the presence of the hydrated hydrogel matrix may serve the double purpose of supporting cell colonization by reducing the hydrophobicity of the scaffolds.

Overall, this system holds great promise in TERM applications, warranting its further investigation in future studies.

## Figures and Tables

**Table 1:** Poly(urethane) (PU) nomenclature and compositional information.

Composition			Nomenclature
Chain Extender	Diisocyanate	Macrodiol	
l-lysine ethyl ester (K)	1,6-hexamethylene diisocyanate (H)	Poly(ε-caprolactone) diol (C2000)	KHC2000
*N*-Boc serinol (N)	1,6-hexamethylene diisocyanate (H)	Poly(ε-caprolactone) diol (C2000)	NHC2000
*N*-Boc serinol (N)	1,6-hexamethylene diisocyanate (H)	Poloxamer^®^ 407 (P407)	NHP407

**Table 2:** Number average molecular weight ($\bar{M_{n}}$) and molecular weight distribution (i.e., polydispersity index $\bar{M_{w}}/\bar{M_{n}}$) for KHC2000, NHC2000, and NHP407 poly(urethane)s.

Sample	$\bar{M_{n}}\;(\mathrm{Da})$	$\bar{M_{w}}/\bar{M_{n}}$	Water Contact Angle
KHC2000	6 × 10^4^	1.6	88 ± 0.4
NHC2000	4 × 10^4^	1.4	75 ± 1
NHP407	5 × 10^4^	1.3	-

**Table 3:** Young’s modulus, and strain and stress at break for KHC2000 scaffolds characterized through tensile tests in dry and wet conditions at room temperature.

Testing Conditions	Young’s Modulus (MPa)		Stress at Break (MPa)		Strain at Break (%)	
	Longitudinal	Cross Section	Longitudinal	Cross Section	Longitudinal	Cross Section
DRY	2.7 ± 0.7	0.8 ± 0.2	0.6 ± 0.2	0.3 ± 0.04	170 ± 19	148 ± 14
WET	1.0 ± 0.3	-	0.4 ± 0.05	-	186 ± 38	-

**Table 4:** Tube-inverting and rheological test results for NHP407 and NHP407_NPs hydrogels.

Parameters		NHP407	NHP407_NPs
Tube-inverting test	LCGT (°C)	32 °C	29 °C
	Gelation time at 37 °C (min)	7 min	7 min
Strain sweep test	γ_L_ (%)	18.6%	29.7%
	γ_gel-sol_ (%)	35%	70%
	T_onset_ (°C)	20.7 °C	19.4 °C
Temperature ramp test	Initial viscosity (Pa·s)	0.15 Pa·s	0.26 Pa·s
	Minimum viscosity (Pa·s)	0.06 Pa·s	0.11 Pa·s
	Viscosity @ 25 °C (Pa·s)	22.58 Pa·s	5.64 Pa·s
Frequency sweep test	ω_CROSS_ at 25 °C (rad/s)	>100 rad/s	>100 rad/s
	ω_CROSS_ at 30 °C (rad/s)	10 rad/s	12.9 rad/s
	ω_CROSS_ at 37 °C (rad/s)	0.33 rad/s	0.29 rad/s

## Data Availability

The data presented in this study are available upon request from the corresponding author.

## References

[1] Salgado A.J., Oliveira J.M., Martins A., Teixeira F.G., Silva N.A., Neves N.M., Sousa N., Reis R.L. (2013). Tissue engineering and regenerative medicine. Int. Rev. Neurobiol..

[2] Frey B.M., Zeisberger S.M., Hoerstrup S.P. (2016). Tissue engineering and regenerative medicine-New initiatives for individual treatment offers. Transfus. Med. Hemother..

[3] Kim M., Evans D. (2005). Tissue engineering: The future of stem cells. Top. Tissue Eng..

[4] Caddeo S., Boffito M., Sartori S. (2017). Tissue engineering approaches in the design of healthy and pathological in vitro tissue models. Front. Bioeng. Biotechnol..

[5] Silvestri A., Boffito M., Sartori S., Ciardelli G. (2013). Biomimetic materials and scaffolds for myocardial tissue regeneration. Macromol. Biosci..

[6] Boffito M., Sartori S., Ciardelli G. (2013). Polymeric scaffolds for cardiac tissue engineering: Requirements and fabrication technologies. Polym. Int..

[7] Kai D., Prabhakaran M.P., Jin G., Ramakrishna S. (2011). Guided orientation of cardiomyocytes on electrospun aligned nanofibers for cardiac tissue engineering. J. Biomed. Mater. Res. Part B Appl. Biomater..

[8] Parrag I.C., Zandstra P.W., Woodhouse K.A. (2011). Fiber alignment and coculture with fibroblasts improves the differentiated phenotype of murine embryonic stem cell-derived cardiomyocytes for cardiac tissue engineering. Biotechnol. Bioeng..

[9] Andersson K.-E., Christ G.J. (2007). Regenerative pharmacology: The future is now. Mol. Interv..

[10] Christ G.J., Saul J.M., Furth M.E., Andersson K.-E. (2013). The pharmacology of regenerative medicine. Pharmacol. Rev..

[11] Fu K., Klibanov A.M., Langer R. (2000). Protein stability in controlled-release systems. Nat. Biotechnol..

[12] Kumari A., Yadav S.K., Yadav S.C. (2010). Biodegradable polymeric nanoparticles based drug delivery systems. Colloids Surf. B..

[13] Pelss J., Loca D., Berzina-Cimdina L., Locs J., Lakevics V. (2011). Release of anticancer drug doxorubicin from biodegradable polymer coated porous hydroxyapatite scaffolds. Adv. Mater. Res..

[14] Wang H., Deng Z., Chen J., Qi X., Pang L., Lin B., Adib Y.T.Y., Miao N., Wang D., Zhang Y. (2020). A novel vehicle-like drug delivery 3D printing scaffold and its applications for a rat femoral bone repairing in vitro and in vivo. Int. J. Biol. Sci..

[15] Dai J., Jin J., Yang S., Li G. (2017). Doxorubicin-loaded PLA/pearl electrospun nanofibrous scaffold for drug delivery and tumor cell treatment. Mater. Res. Express.

[16] Zafar M., Najeeb S., Khurshid Z., Vazirzadeh M., Zohaib S., Najeeb B., Sefat F. (2016). Potential of electrospun nanofibers for biomedical and dental applications. Materials.

[17] Qasim S.B., Zafar M.S., Najeeb S., Khurshid Z., Shah A.H., Husain S., Rehman I.U. (2018). Electrospinning of chitosan-based solutions for tissue engineering and regenerative medicine. Int. J. Mol. Sci..

[18] Husain S., Al-Samadani K.H., Najeeb S., Zafar M.S., Khurshid Z., Zohaib K., Qasim S.B. (2017). Chitosan biomaterials for current and potential dental applications. Materials.

[19] Sarwar M.S., Huang Q., Ghaffar A., Abid M.A., Zafar M.S., Khurshid Z., Latif M. (2020). A smart drug delivery system based on biodegradable chitosan/Poly(Allylamine hydrochloride) blend films. Pharmaceutics.

[20] Çelebier S.K., Bozdağ K., Pehlivan S.B., Demirbilek M., Akıncı M., Vural I., Akdağ Y., Yürüker S., Ünlü N. (2020). Development of an anti-inflammatory drug-incorporated biomimetic scaffold for corneal tissue engineering. J. Ocul. Pharmacol. Ther..

[21] Hsin-Yi L., Tsang-Wen C. (2016). Chitosan-coated alginate tissue scaffold constructed by three-dimensional plotting technique used for drug and cell delivery. Front. Bioeng. Biotechnol..

[22] Brachi G., Ruiz-Ramírez J., Dogra P., Wang Z., Cristini V., Ciardelli G., Rostomily R.C., Ferrari M., Mikheev A.M., Blanco E. (2020). Intratumoral injection of hydrogel-embedded nanoparticles enhances retention in glioblastoma. Nanoscale.

[23] Dewhurst R.M., Scalzone A., Buckley J., Mattu C., Rankin K.S., Gentile P., Ferreira A.M. (2020). Development of natural-based bone cement for a controlled doxorubicin-drug release. Front. Bioeng. Biotechnol..

[24] Boffito M., Laurano R., Giasafaki D., Steriotis T., Papadopoulos A., Tonda-Turo C., Cassino C., Charalambopoulou G., Ciardelli G. (2020). Embedding ordered mesoporous carbons into thermosensitive hydrogels: A cutting-edge strategy to vehiculate a cargo and control its release profile. Nanomaterials.

[25] Pontremoli C., Boffito M., Fiorilli S., Laurano R., Torchio A., Bari A., Tonda-Turo C., Ciardelli G., Vitale-Brovarone C. (2018). Hybrid injectable platforms for the in situ delivery of therapeutic ions from mesoporous glasses. Chem. Eng. J..

[26] Shokry H., Mattinen U., Wiltschka O., Niinimäki J., Lerche M., Levon K., Lindén M., Sahlgren C. (2015). Mesoporous silica particle-PLA–PANI hybrid scaffolds for cell-directed intracellular drug delivery and tissue vascularization. Nanoscale.

[27] He D., Zhao A., Su H., Zhang Y., Wang Y., Luo D., Gao Y., Li J., Yang P. (2019). An injectable scaffold based on temperature-responsive hydrogel and factor-loaded nanoparticles for application in vascularization in tissue engineering. J. Biomed. Mater. Res. Part A.

[28] Asghar W., Islam M., Wadajkar A.S., Wan Y., Ilyas A., Nguyen K.T., Iqbal S.M. (2012). PLGA micro-and nanoparticles loaded into gelatin scaffold for controlled drug release. IEEE Trans. Nanotechnol..

[29] Nooeaid P., Chuysinuan P., Pengsuk C., Dechtrirat D., Lirdprapamongkol K., Techasakul S., Svasti J. (2020). Polylactic acid microparticles embedded porous gelatin scaffolds with multifunctional properties for soft tissue engineering. J. Sci. Adv. Mater. Devices.

[30] Gentile P., Nandagiri V.K., Daly J., Chiono V., Mattu C., Tonda-Turo C., Ciardelli G., Ramtoola Z. (2016). Localised controlled release of simvastatin from porous chitosan–gelatin scaffolds engrafted with simvastatin loaded PLGA-microparticles for bone tissue engineering application. Mater. Sci. Eng. C.

[31] Gentile P., Bellucci D., Sola A., Mattu C., Cannillo V., Ciardelli G. (2015). Composite scaffolds for controlled drug release: Role of the polyurethane nanoparticles on the physical properties and cell behaviour. J. Mech. Behav. Biomed. Mater..

[32] Ferreira A.M., Mattu C., Ranzato E., Ciardelli G. (2014). Bioinspired porous membranes containing polymer nanoparticles for wound healing. J. Biomed. Mater. Res. Part A.

[33] Smith I.O., Liu X.H., Smith L.A., Ma P.X. (2009). Nanostructured polymer scaffolds for tissue engineering and regenerative medicine. Wiley Interdiscip. Rev. Nanomed. Nanobiotechnol..

[34] Sartori S., Boffito M., Serafini P., Caporale A., Silvestri A., Bernardi E., Sassi M.P., Boccafoschi F., Ciardelli G. (2013). Synthesis and structure–property relationship of polyester-urethanes and their evaluation for the regeneration of contractile tissues. React. Funct. Polym..

[35] Gioffredi E., Boffito M., Calzone S., Giannitelli S.M., Rainer A., Trombetta M., Mozetic P., Chiono V. (2016). Pluronic F127 hydrogel characterization and biofabrication in cellularized constructs for tissue engineering applications. Procedia CIRP.

[36] Mattu C., Pabari R., Boffito M., Sartori S., Ciardelli G., Ramtoola Z. (2013). Comparative evaluation of novel biodegradable nanoparticles for the drug targeting to breast cancer cells. Eur. J. Pharm. Biopharm..

[37] Riboldi S.A., Sampaolesi M., Neuenschwander P., Cossu G., Mantero S. (2005). Electrospun degradable polyesterurethane membranes: Potential scaffolds for skeletal muscle tissue engineering. Biomaterials.

[38] Levy-Mishali M., Zoldan J., Levenberg S. (2009). Effect of scaffold stiffness on myoblast differentiation. Tissue Eng. Part A.

[39] Boffito M., Gioffredi E., Chiono V., Calzone S., Ranzato E., Martinotti S., Ciardelli G. (2016). Novel polyurethane-based thermosensitive hydrogels as drug release and tissue engineering platforms: Design and in vitro characterization. Polym. Int..

[40] Boffito M., Bernardi E., Sartori S., Ciardelli G., Sassi M.P. (2014). A mechanical characterization of polymer scaffolds and films at the macroscale and nanoscale. J. Biomed. Mater. Res. Part A.

[41] Guan J., Fujimoto K.L., Sacks M.S., Wagner W.R. (2005). Preparation and characterization of highly porous, biodegradable polyurethane scaffolds for soft tissue applications. Biomaterials.

[42] Bhattacharya M., Malinen M.M., Lauren P., Lou Y.-R., Kuisma S.W., Kanninen L., Lille M., Corlu A., GuGuen-Guillouzo C., Ikkala O. (2012). Nanofibrillar cellulose hydrogel promotes three-dimensional liver cell culture. J. Control. Release.

[43] Brand-Williams W., Cuvelier M., Berset C. (1995). Use of a free radical method to evaluate antioxidant activity. LWT Food Sci. Technol..

[44] Rahman M., Islam B., Biswas M., Alam A.H.M.K. (2015). In vitro antioxidant and free radical scavenging activity of different parts of Tabebuia pallida growing in Bangladesh. BMC Res. Notes.

[45] Laurano R., Abrami M., Grassi M., Ciardelli G., Boffito M., Chiono V. (2020). Using poloxamer® 407 as building block of amphiphilic poly(Ether urethane)S: Effect of its molecular weight distribution on thermo-sensitive hydrogel performances in the perspective of their biomedical application. Front. Mater..

[46] Areias A.C., Ribeiro C., Sencadas V., Garciagiralt N., Diezperez A., Ribelles J.L.G., Lanceros-Méndez S. (2012). Influence of crystallinity and fiber orientation on hydrophobicity and biological response of poly(l-lactide) electrospun mats. Soft Matter.

[47] Herzog K., Müller R.-J., Deckwer W.-D. (2006). Mechanism and kinetics of the enzymatic hydrolysis of polyester nanoparticles by lipases. Polym. Degrad. Stab..

[48] Boffito M., Di Meglio F., Vitale N., Brancaccio M., Tarone G., Basoli F., Rainer A., Trombetta M., Ciardelli G., Chiono V. (2018). Surface functionalization of polyurethane scaffolds mimicking the myocardial microenvironment to support cardiac primitive cells. PLoS ONE.

[49] Klouda E.L., Vaz C.M., Driessen-Mol A.A., Baaijens F.P.T., Bouten C.V.C. (2007). Effect of biomimetic conditions on mechanical and structural integrity of PGA/P4HB and electrospun PCL scaffolds. J. Mater. Sci. Mater. Med..

[50] Pan Z., Ding J. (2012). Poly(lactide- co -glycolide) porous scaffolds for tissue engineering and regenerative medicine. Interface Focus.

[51] Zhou Q., Gong Y., Gao C. (2005). Microstructure and mechanical properties of poly(L-lactide) scaffolds fabricated by gelatin particle leaching method. J. Appl. Polym. Sci..

[52] Anitha A., Deepagan V., Rani V.D., Menon D., Nair S., Jayakumar R. (2011). Preparation, characterization, in vitro drug release and biological studies of curcumin loaded dextran sulphate–chitosan nanoparticles. Carbohydr. Polym..

[53] Esfandiarpour-Boroujeni S., Bagheri-Khoulenjani S., Mirzadeh H., Amanpour S. (2017). Fabrication and study of curcumin loaded nanoparticles based on folate-chitosan for breast cancer therapy application. Carbohydr. Polym..

[54] Iodice C., Cervadoro A., Palange A., Key J., Aryal S., Ramirez M.R., Mattu C., Ciardelli G., O’Neill B.E., Decuzzi P. (2016). Enhancing photothermal cancer therapy by clustering gold nanoparticles into spherical polymeric nanoconstructs. Opt. Lasers Eng..

[55] Mattu C., Boffito M., Sartori S., Ranzato E., Bernardi E., Sassi M.P., Di Rienzo A.M., Ciardelli G. (2012). Therapeutic nanoparticles from novel multiblock engineered polyesterurethanes. J. Nanopart. Res..

[56] Rejinold N.S., Muthunarayanan M., Divyarani V., Sreerekha P., Chennazhi K., Nair S., Tamura H., Jayakumar R. (2011). Curcumin-loaded biocompatible thermoresponsive polymeric nanoparticles for cancer drug delivery. J. Colloid Interface Sci..

[57] Kulisic T., Radonic A., Katalinic V., Milos M. (2004). Use of different methods for testing antioxidative activity of oregano essential oil. Food Chem..

[58] Aoki T., Kawashima M., Katono H., Sanui K., Ogata N., Okano T., Sakurai Y. (1994). Temperature-responsive interpenetrating polymer networks constructed with poly(Acrylic acid) And poly(N,n-dimethylacrylamide). Macromolecules.

[59] Boffito M., Torchio A., Schmidt-Bleek K., Ciardelli G., Tonda-Turo C., Laurano R., Gisbert-Garzarán M., Berkmann J.C., Cassino C., Manzano M. (2020). Hybrid injectable Sol-gel systems based on thermo-sensitive polyurethane hydrogels carrying PH-sensitive mesoporous silica nanoparticles for the controlled and triggered release of therapeutic agents. Front. Bioeng. Biotechnol..

[60] Esmaili Z., Bayrami S., Dorkoosh F.A., Javar H.A., Seyedjafari E., Zargarian S.S., Haddadi-Asl V. (2017). Development and characterization of electrosprayed nanoparticles for encapsulation of Curcumin. J. Biomed. Mater. Res. Part A.

[61] Sivasami P., Hemalatha T. (2018). Augmentation of therapeutic potential of curcumin using nanotechnology: Current perspective. Artif. Cells Nanomed. Biotechnol..

[62] Naksuriya O., Van Steenbergen M.J., Torano J.S., Okonogi S., Hennink W.E. (2016). A kinetic degradation study of curcumin in its free form and loaded in polymeric micelles. AAPS J..

